# Membrane Filtration of Sonication Fluid—A Promising Adjunctive Method for the Diagnosis of Low‐Grade Infection in Presumed Aseptic Nonunion

**DOI:** 10.1002/jor.26076

**Published:** 2025-03-24

**Authors:** Katharina Trenkwalder, Sandra Erichsen, Ferdinand Weisemann, Peter Augat, SAND Research Group, Simon Hackl, Matthias Militz, Simon Hackl, Ferdinand Weisemann, Katharina Trenkwalder, Sandra Erichsen, Tobias Hentschel, Peter Augat, Heiko Baumgartner, Marie Reumann, Georg Reiter, Holger Freischmidt, Matthias Kemmerer, Steffen Langwald, John Hanke, Martin Glombitza, Eva Steinhausen, Ulf‐Joachim Gerlach, Nikolai Spranger, Dirk Stengel

**Affiliations:** ^1^ Institute for Biomechanics BG Unfallklinik Murnau Murnau am Staffelsee Germany; ^2^ Institute for Biomechanics Paracelsus Medical University Salzburg Austria; ^3^ Department of Trauma Surgery BG Unfallklinik Murnau Murnau am Staffelsee Germany; ^4^ BG Unfallklinik Murnau Murnau am Staffelsee Germany; ^5^ BG Klinik Tübingen Tübingen Germany; ^6^ BG Klinik Ludwigshafen Ludwigshafen Germany; ^7^ BG Unfallklinik Frankfurt Frankfurt Germany; ^8^ BG Klinikum Bergmannstrost Halle Halle Germany; ^9^ BG Klinikum Duisburg Duisburg Germany; ^10^ BG Klinikum Hamburg Hamburg Germany; ^11^ BG Klinikum Unfallkrankenhaus Berlin Berlin Germany; ^12^ BG Kliniken – Klinikverbund Berlin Germany

**Keywords:** fracture‐related infection, implant sonication, low‐grade infected nonunion, membrane filtration, suspected aseptic nonunion

## Abstract

Treatment guidelines for fracture nonunion differ based on the presence or absence of infection. Low‐grade infections without preoperative clinical signs of infection are difficult to distinguish from aseptic cases. Membrane filtration of sonication fluid (MF) has been shown to be a useful method for identifying septic nonunion. Therefore, the aim of this study was to evaluate the diagnostic value of MF in differentiating low‐grade infected nonunion from aseptic cases. A prospective multicenter clinical study enrolled 75 patients with femoral or tibial shaft nonunion with planned revision surgery and without clinical suspicion of infection. During revision surgery, tissue from the nonunion zone was sampled for culture and histopathology, and the implant for sonication with MF and colony forming unit (CFU) quantification. Infection was diagnosed according to the diagnostic criteria for fracture‐related infection. The diagnostic performance of MF CFU count was evaluated by receiver operating characteristic (ROC) curve and compared with that of tissue culture (TC), sonication fluid broth culture (SFC), and Histopathological Osteomyelitis Evaluation Score (HOES). Fifty‐three nonunion cases were aseptic, and 22 had a low‐grade infection. ROC curve had an area under the curve of 0.84. The optimal CFU cutoff to discriminate between low‐grade infected and aseptic nonunion was 11.1 CFU/10 mL sonication fluid with 64% sensitivity and 89% specificity. SFC showed a higher sensitivity of 82% but a lower specificity of 81%. The sensitivity and specificity of TC were 77% and 96%, respectively, and those of HOES were 9% and 87%, respectively. Implementation of MF in clinical diagnostics as an adjunct to TC may improve the differential diagnosis between low‐grade infected nonunion and aseptic nonunion.

## 1 Introduction

Treatment guidelines for fracture nonunion diverge based on the presence or absence of bacterial infection [1, 2]. Infection in nonunion is diagnosed using preoperative and intraoperative criteria for the diagnosis of fracture‐related infection (FRI) [3]. In the absence of preoperative clinical signs of infection, aseptic nonunion is often assumed, and diagnosis or exclusion of infection is mainly based on intraoperative samples obtained during revision surgery for microbiology and histopathology [4]. An infection detected by intraoperative diagnostics in the absence of any preoperative indication of infection is referred to as a low‐grade infection [5, 6]. A recently published systematic review of 21 studies of presumed aseptic long‐bone nonunion revealed a low‐grade infection rate of 10% (range between studies: 0%–37%) [4]. Because of this substantial prevalence of low‐grade infection in nonunion and the associated need for systemic antimicrobial therapy, it is important to be able to reliably identify them. However, sensitivities for FRI detection by long‐term tissue culture (TC) or quantitative histology are modest and range from 60% to 89% [7, 8, 9, 10, 11] and from 14% to 80% [10, 11, 12, 13], respectively. Thus, there is a need for novel methods to complement current diagnostics to improve the diagnosis of infection in nonunion.

Low‐grade infections are often caused by low‐virulent bacterial species such as coagulase‐negative staphylococci (CoNS) or *Cutibacterium acnes* [4]. These microorganisms are able to attach to the surface of the implant, aggregate irreversibly, and form biofilms that are difficult to treat due to poor response to antibiotics [14, 15]. Biofilm‐embedded microorganisms can be dislodged from the implant by sonication, followed by analysis of the sonication fluid to identify bacterial species [16]. Sonication has become an important element in the diagnosis of peri‐prosthetic joint infection (PJI) [16] and might be a useful adjunct in diagnosing FRI. Only a few studies have focused on implant sonication for the diagnosis of FRI alone, without also including PJI cases [7, 9, 13]. The laboratory protocols for sonication in these studies were heterogeneous, but their results indicated the potential of sonication as an adjunct to TC in increasing sensitivity [7, 13] and aiding in the detection of polymicrobial infections [9, 13]. However, due to this lack of scientific evidence specific to FRI, the true added value of sonication in the diagnosis of FRI is still unclear [3, 17].

There are several methods for analyzing the sonication fluid, including enrichment broth cultures or plating on agar with colony forming unit (CFU) quantification with or without prior concentration by centrifugation [18]. In a previous study by the authors, a novel method of membrane filtration of sonication fluid (MF) with quantification of CFU was introduced as a promising diagnostic tool for the identification of septic nonunion. In that study, this approach was evaluated in a cohort of patients with septic and aseptic nonunion, using a calculated optimal CFU cutoff to distinguish between the two conditions. Advantages of the novel technique over TC and sonication fluid broth culture (SFC) included an earlier availability of the results and a higher polymicrobial detection rate [13]. However, the previous study included both patients with and without preoperative clinical signs of infection, and it is conceivable that cases with clinically inconspicuous low‐grade infections have different bacterial loads on the implant compared to cases with infections that were clinically evident before surgery. Therefore, the aim of the present study was to investigate within a subset of the patient cohort from the previous study, whether MF is still a suitable method for differentiating cases with low‐grade infection from aseptic cases in nonunion patients who lack preoperative clinical signs of infection, a scenario that poses a significant challenge in clinical practice.

## 2 Patients and Methods

### 2.1 Study Population

This prospective multicenter clinical study investigated the microbiological colonization of femoral and tibial fixation material in aseptic and low‐grade infected nonunion. Patients with femoral or tibial shaft nonunion and presumed aseptic genesis aged ≥ 18 years were prospectively identified at seven level I trauma centers in Germany between January 2019 and November 2021. The level of evidence II study was approved by the Ethics Committee of the Institutional and National Medical Board (Bavarian State Chamber of Physicians, ID 2016‐16041) and registered in the German Clinical Trials Register (DRKS00014657). Each study center received an ethics vote from its locally responsible ethics committee. Seventy‐five patients who were admitted to the hospitals for revision surgery gave written consent and were included in this study. Nonunion was defined as a fracture that does not heal without further surgical intervention, independent of the length of the previous treatment [19]. The diagnosis of nonunion was based on the patient's complaints, clinical examination, and mandatory conventional radiographs. Radiologic signs of nonunion were defined as a lack of osseous bridging in at least three out of four cortices assessed on anteroposterior and lateral views of conventional radiographs [20]. Before revision surgery, medical history, clinical appearance, and blood count, including the C‐reactive protein of the patients, gave no indication of infection as an underlying cause of nonunion. Therefore, an aseptic nonunion was suspected. Exclusion criteria were suspicion of an underlying infection, the administration of preoperative long‐term antibiotics or current antimicrobial therapy, and pregnancy. Only patients with complete implant replacement were involved in this investigation. The patients were followed for 12 months after the initial surgical nonunion revision.

### 2.2 Sample Collection

The nonunion revision was performed according to clinical standards. Besides the clinical specimens for microbiology and histopathology, which were analyzed at each study site according to their own standard clinical operating procedures, two additional study tissue samples were obtained from the transition between healthy bone and nonunion. These samples were directly transferred into 9 mL sterile thioglycolate broth with resazurin (bioMérieux) for conventional microbiological examination or 4% phosphate‐buffered formaldehyde solution (AppliChem) for histopathological examination. Osteosynthesis material was removed under sterile conditions according to clinical standards and transferred into sterile stand‐up bags (Whirl‐Pak, Nasco Sampling). All study samples collected during revision surgery were transported overnight to the Institute for Biomechanics at the BG Unfallklinik Murnau and were processed on the subsequent day.

### 2.3 Sample Analysis

**Figure 1:**
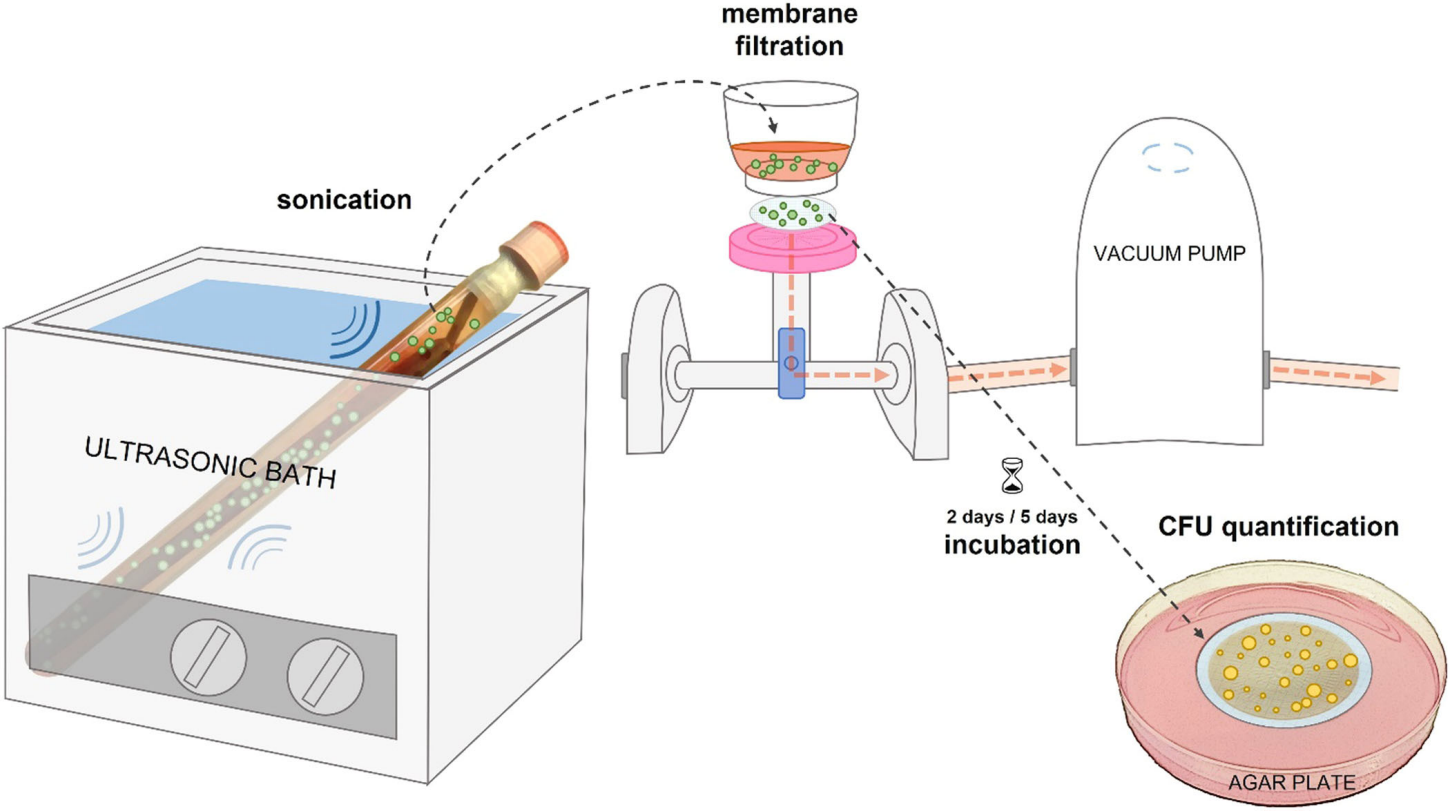
Membrane filtration of sonication fluid—Schematic overview of the diagnostic method. CFU, colony forming unit.

Sonication and MF were performed as described previously [13]. In brief, sonication was conducted in 150 mL Ringer's solution (Ringer Fresenius, Fresenius Kabi Deutschland GmbH) with the entire intramedullary nail or locking plate; screws were included if provided. Four vacuum filtrations with 20 mL sonication fluid each were performed through membrane filters with a pore size of 0.45 μm (EZ‐Fit, Millipore, Merck KGaA). The filter membranes were incubated on Columbia agar plates with 5% sheep blood (Oxoid Germany GmbH, Thermo Fisher Scientific), two under aerobic conditions for at least 2 days (median [IQR]: 3 [2–4]) and two under anaerobic conditions generated with an Anoxomat system (Advanced Instruments, Norwood, the United States) for at least 5 days (median [IQR]: 5 [5–5]), with variations due to weekends and holidays. A schematic overview of this method is given in Figure 1.

Incubation was followed by CFU quantification on all four membrane filters. After quantification, either the two aerobically incubated or the two anaerobically incubated filter membranes were selected for evaluation, whichever culture condition yielded the higher CFU counts according to Trampuz et al. to avoid bias in the presence of obligate anaerobic bacteria [21]. Colonies were considered only if at least one other colony of the same pathogen grew on the selected membrane filters. Single colonies were considered as contaminants. Counts greater than 250 CFU per membrane filter were recorded as too numerous to count (TNTC). On Columbia agar with 5% sheep blood (Oxoid Germany GmbH, Thermo Fisher Scientific), morphologically distinct colony types were isolated and then identified using matrix‐assisted laser desorption ionization time‐of‐light (MALDI‐TOF) mass spectrometry to species level (Vitek MS, bioMérieux Vitek Inc.). Identified pathogens were tested for antibiotic susceptibility (Vitek 2, bioMérieux Vitek Inc.). As laboratory‐negative controls, eight sterile intramedullary nails and plates were transferred into sterile stand‐up bags (Whil‐Pak, Nasco Sampling) and tested in the same manner.

The study TC samples were incubated at 37°C and 5% CO_2_ for 14 days. Additionally, 5 mL of the sonication fluid was inoculated into 9 mL thioglycolate broth with resazurin (bioMérieux) and incubated at 37°C and 5% CO_2_ for 10 days as a diagnostic implant specimen. After steaking out on Columbia agar with 5% sheep blood (Oxoid Germany GmbH, Thermo Fisher Scientific), grown bacterial colonies of tissue and implant samples were identified and tested for antibiotic susceptibility analogously to MF.

Tissue samples fixed in formaldehyde for histopathology were decalcified and stained with H&E staining, Berlin blue, and PAS reaction after sectioning. Polarization optical analysis was performed to semi‐quantitatively evaluate the sections for signs of osteomyelitis using the Histopathological Osteomyelitis Evaluation Score (HOES) [22].

### 2.4 Infection Diagnosis

The diagnosis of “aseptic” or “low‐grade‐infected” nonunion was made according to the FRI consensus definition [23]. The microbiological and histopathological findings from the initial revision surgery or from any follow‐up surgery within 12 months of study inclusion were considered for diagnosis. A low‐grade infection was confirmed by the detection of identical pathogens by culture from at least two separate deep tissue or implant specimens, or by the detection of microorganisms in a TC with the concomitant presence of histopathological signs of infection. The results of the MF were not taken into account as a diagnostic criterion, as the evaluation of this method was the subject of the present study.

### 2.5 Statistics

Statistical analyses were conducted in SPSS (ver. 26; IBM). After the normal distributions of the metric variables within groups were examined graphically using histograms and Q–Q plots, group comparisons were made using *t*‐tests for normal distributions and Mann–Whitney *U* tests for nonnormally distributed variables. Qualitative variables were compared with Pearson's *χ* ^2^ or Fisher's exact test. A *p* value < 0.05 (two‐sided) was considered statistically significant. In the case of 2 × 2 contingency tables, *p* values of Pearson's *χ* ^2^ test were corrected for continuity according to Yates, or *p* values of the one‐tailed Fisher test were doubled. CFU cutoff for infection diagnosis based on the MF method was calculated with the receiver operating characteristic (ROC) curve and the Youden Index. 2 × 2 tables were used to determine the diagnostic performance of MF and, for comparison, the conventional diagnostic methods of TC and HOES, as well as the SFC. 95% Confidence intervals (CIs) for sensitivity, specificity, and positive and negative predictive values (PPV and NPV) were generated via Clinical Calculator 1 (http://vassarstats.net/ [November 11, 2024]).

## 3 Results

Low‐grade infection was diagnosed in 22 patients according to the above criteria, while 53 patients had an aseptic nonunion, forming the two study groups for further analysis. These two groups were not statistically different in terms of sex, age, affected bone, or soft tissue injury. The only significant difference was a higher body mass index (BMI) in the low‐grade infected nonunion group. There were no significant differences in intraoperative sampling for diagnostics between both groups (Table 1).

**Table 1:** Patient and diagnostic sample characteristics of the study groups.

Diagnosis	*n* (%)		
Patient and sample characteristics	Aseptic nonunion (*n* = 53)	Low‐grade infected nonunion (*n* = 22)	*p* value
Sex			0.127
Male	32 (60%)	18 (82%)	
Female	21 (40%)	4 (18%)	
Age; mean (SD) [years]	48 (15)	43 (12)	0.118
BMI; mean (SD)	27 (5)	30 (6)	0.032*
Bone			0.649
Tibia	29 (55%)	14 (64%)	
Femur	24 (45%)	8 (36%)	
Fracture			1.000
Open	25 (47%)	11 (50%)	
Closed	28 (53%)	11 (50%)	
Osteosynthesis material for sonication			0.071
Intramedullary nail	40 (76%)	13 (59%)	
Plate	13 (25%)	7 (32%)	
Intramedullary nail + plate	0 (0%)	2 (9%)	
Number of collected tissue samples for			
TC; median (IQR)	3 (2–4)	4 (2–5)	0.124
Histopathology; median (IQR)	1 (1–2)	1 (1–2)	0.843

Abbreviations: BMI, body mass index; IQR, interquartile range; SD, standard deviation; TC, tissue culture.

* *p* < 0.05.

### 3.1 Infection Diagnosis by MF and Conventional Diagnostics

**Figure 2:**
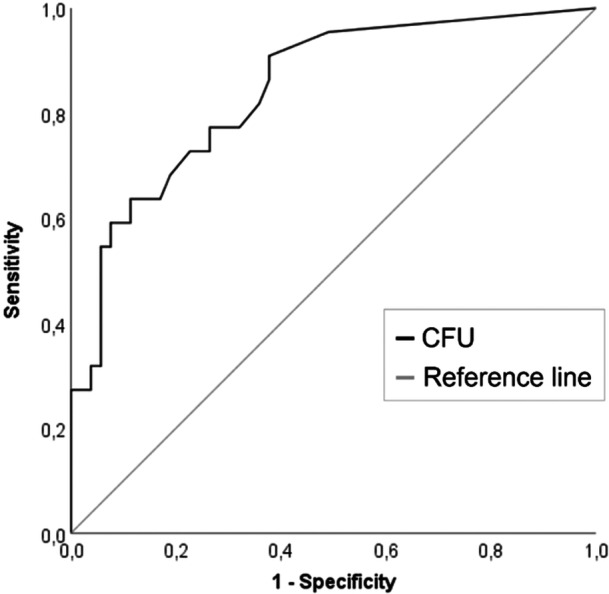
Receiver operating characteristic curve for the discrimination of aseptic and low‐grade infected nonunion on the basis of the number of colony forming unit (CFU) per 10 mL sonication fluid.

For patients with aseptic nonunion, MF CFU quantification revealed a median of 0 (IQR: 0–3) CFU per 10 mL sonication fluid, while low‐grade infection was characterized by a median of 21 (IQR: 3‐TNTC) CFU per 10 mL sonication fluid (*p* < 0.001). ROC analysis of CFU demonstrated an area under the curve (AUC) of 0.84 (95% CI 0.75–0.94; Figure 2).

The highest Youden Index (0.53) was found for 0.75 CFU per 10 mL sonication fluid with a sensitivity of 91% and specificity of 62%. However, because high specificity takes precedence over an optimal trade‐off between sensitivity and specificity [21], 11.1 CFU per 10 mL sonication fluid, which had the second highest Youden Index (0.52), was selected as the optimal CFU cutoff value to discriminate between aseptic nonunion and low‐grade infected nonunion. Using this cutoff, low‐grade infected nonunion could be diagnosed with moderate sensitivity (64%) and high specificity (89%). Long‐term TC demonstrated the best diagnostic performance, while the sensitivity of histopathology was extremely low. SFC had a high sensitivity; however, this method demonstrated the lowest specificity. The performance of all diagnostic methods is given in Table 2.

**Table 2:** Performances of diagnostic methods in the study surgery.

	No. of test‐positive patients		% (95% CI)			
Test method	Low‐grade infected nonunion	Aseptic nonunion	Sensitivity	Specificity	PPV	NPV
TC (study sample)	17/22	2/53	77 (54–91)	96 (86–99)	90 (66–98)	91 (80–97)
HOES (study sample)	2/22	7/53	9 (2–31)	87 (74–94)	22 (4–60)	70 (57–80)
SFC	18/22	10/53	82 (59–94)	81 (68–90)	64 (44–81)	92 (79–97)
MF (≥ 11.1 CFU/10 mL)	14/22	6/53	64 (41–82)	89 (76–95)	70 (46–87)	86 (73–93)

Abbreviations: CFU, colony forming unit; CI, confidence interval; HOES, Histopathological Osteomyelitis Evaluation Score; MF, membrane filtration of sonication fluid; No., number; NPV, negative predictive value; PPV, positive predictive value; SFC, sonication fluid broth culture; TC, tissue culture.

### 3.2 Culture Results of MF in Comparison to TC and SFC

In patients with low‐grade infected nonunion, CoNS and *C. acnes* were the most common pathogens identified by MF, TC, and SFC. There were no significant differences in the bacterial spectrum between the three diagnostic methods (Table 3). However, the methods differed significantly in the number of species detected per sample (Table 3). The number of polymicrobial detections, that is, two or three bacterial species per sample, was significantly different between TC, MF, and SFC (*p* = 0.029), as polymicrobial detection was more frequent with MF (*n* = 8) compared to TC (*n* = 1) (*p* = 0.022).

**Table 3:** Culture results in patients with low‐grade infected nonunion for different diagnostic methods.

Diagnostic method	*n* (%)			
Culture result	TC (study sample)	MF (≥ 11.1 CFU/10 mL)	SFC	*p* value
Number of detected species per sample				0.021*
Negative	5 (23%)	8 (36%)	4 (18%)	
1 species per sample	16 (73%)	6 (27%)	15 (68%)	
2 species per sample	1 (5%)	6 (27%)	3 (14%)	
3 species per sample	0 (0%)	2 (9%)	0 (0%)	
Frequency of detected bacterial species				
*Staphylococcus epidermidis*	5 (23%)	10 (46%)	11 (50%)	0.140
Other CoNS	4 (18%)	7 (32%)	7 (32%)	0.503
*Cutibacterium acnes*	7 (32%)	5 (23%)	2 (9%)	0.215
*Staphylococcus aureus*	2 (9%)	0 (0%)	0 (0%)	0.323
*Enterococcus faecalis*	0 (0%)	1 (5%)	1 (5%)	1.000
*Corynebacterium* spp.	0 (0%)	1 (5%)	0 (0%)	1.000

Abbreviations: CFU, colony forming unit; CoNS, coagulase‐negative staphylococci; MF, membrane filtration of sonication fluid; SFC, sonication fluid broth culture; spp., several species; TC, tissue culture.

* *p* < 0.05.

**Figure 3:**
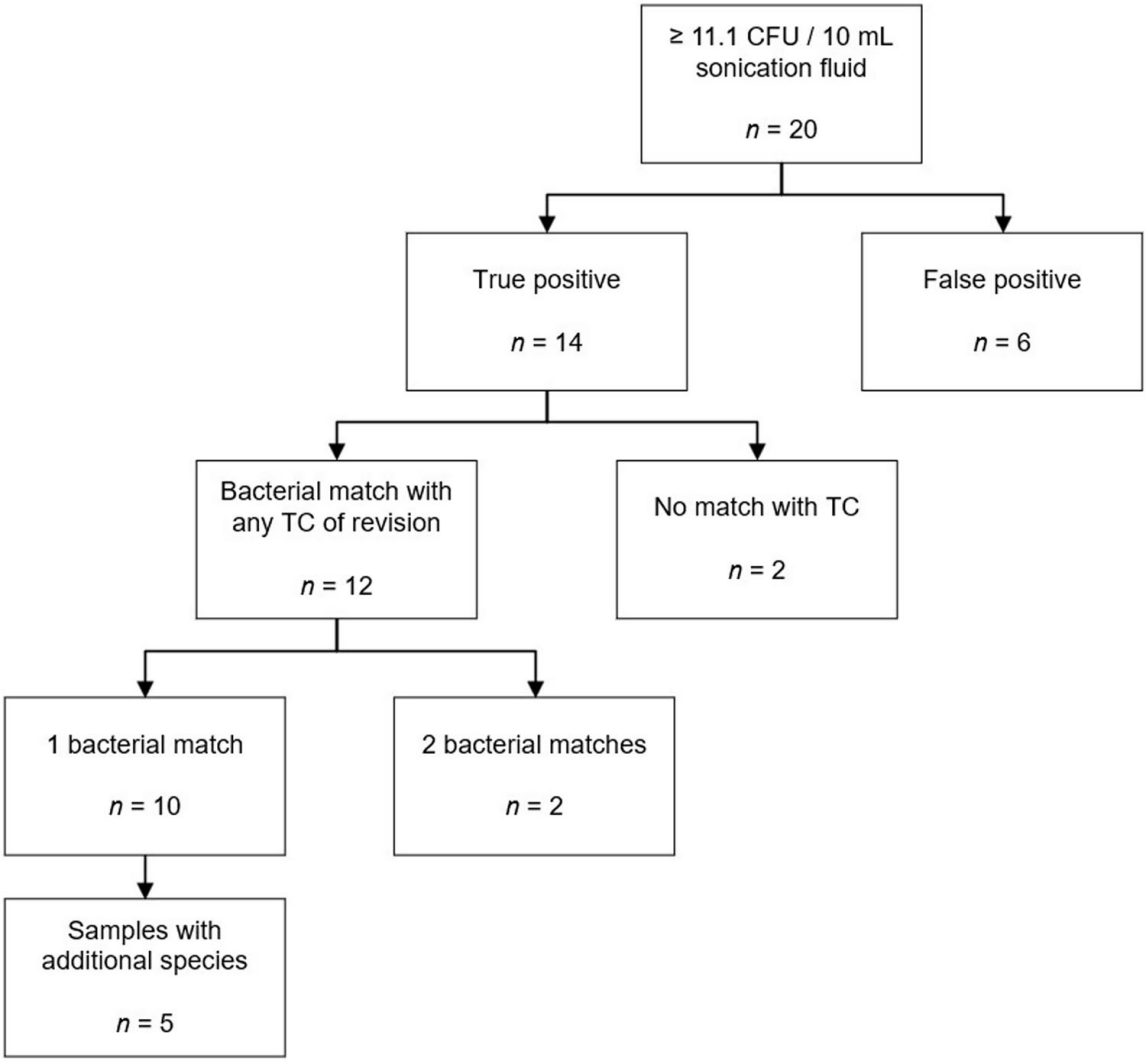
Bacterial detection in patients with nonunion without preoperative suspicion of infection by MF versus TC. This chart contains all MFs performed with CFU counts ≥ 11.1/10 mL sonication fluid. False‐positive cases indicate patients with a CFU count ≥ 11.1/10 mL sonication fluid without a diagnosis of low‐grade infection (aseptic nonunion); true‐positive cases indicate patients with a CFU count ≥ 11.1/10 mL sonication fluid and a diagnosis of low‐grade infected nonunion. Bacterial species detected by MF in true‐positive cases were compared with those of TC for concordance of bacterial species detected (match/no match/additional species). CFU, colony forming unit; MF, membrane filtration of sonication fluid; TC, tissue culture.

Twenty‐seven percentage of the 75 MFs performed were culture positive using the calculated cutoff of 11.1 CFU per 10 mL sonication fluid. Seventy percentage of these were true‐positives, meaning they had a diagnosed infection, whereby 86% were consistent with one or two bacterial species detected in a study or clinical TC of revision surgery; 83% were consistent with a single bacterial species; and 17% were consistent with two bacterial species. Forty‐two percentage of the patients with matching results had additional bacterial detections by MF (Figure 3).

## 4 Discussion

Adequate treatment of fracture nonunion depends on the presence or absence of an infection. However, nonunion with underlying infections that are clinically inconspicuous are common [4]. Therefore, the fundamental difficulty in the clinical practice of nonunion diagnosis is the identification of such low‐grade infections. The aim of the present study was to evaluate the discriminative ability of the novel intraoperative diagnostic method of MF to differentiate infected nonunion from aseptic cases in patients without preoperative indication of infection. Our findings suggest that MF is superior to HOES but inferior to TC in identifying low‐grade infections. The bacterial spectrum did not differ from TC, but MF revealed significantly more polymicrobial infections. In addition, MF culture results are available earlier than with long‐term TC and SFC.

The present study investigated the bacterial load on implants from patients with aseptic and low‐grade infected nonunion by sonication with MF and subsequent CFU quantification. With an AUC exceeding 0.8, the CFU counts of this method demonstrated an excellent discriminatory ability [24] between the two groups. The AUC was higher than that in a previous study by the authors that included patients with preoperative clinical signs of infection and showed an AUC of 0.77 [13], suggesting that MF may be more useful in identifying low‐grade infections. Quantification of the bacterial load in the sonication fluid is important to ensure that only clinically relevant loads on the implant are considered, as bacterial colonization of implants is not necessarily associated with impaired fracture healing or implant‐related infection, as shown in studies with evidence of bacterial colonization of routinely removed implants after regular fracture healing [13, 25]. The higher specificity and the higher PPV of the MF compared to the SFC in this study also emphasize the need for CFU quantification. However, there is still no consensus on a uniform protocol or CFU cutoff for the diagnosis of FRI by sonication [17]. A previous study introducing the MF method examined bacterial colonization rates on implants removed from patients with regularly healed fractures and found that bacterial loads 18.5 times lower than the CFU cutoff value from the present study may be considered as either operating room contamination or clinically irrelevant. In the same study, for the differentiation between septic and aseptic nonunion, the optimal cutoff was slightly higher than in the present study [13]. Apart from this study, the authors are aware of only one other study that calculated a threshold value for the bacterial load in the sonication fluid for the diagnosis of PJI [21]. At 10 CFU per mL sonication fluid, the threshold value in this study was nine times higher than that in the present study. However, the bacterial load on the surface of prostheses in joint infections cannot be compared to that of osteosynthesis material in the case of nonunion. Small‐scale biofilms or bacterial aggregations in the area of the nonunion zone must be considered to be clinically relevant and have a negative impact on fracture healing. To detect such low bacterial loads on the surface of implants, it is necessary to concentrate the sonication fluid. In contrast to the centrifugation of sonication fluid, the advantage of MF used in this study is that a larger volume of sonication fluid can be examined [13]. Although the presence of a mature biofilm on the implant may be questionable in some patients with CFU per 10 mL sonication fluid in the low double digits in the current study, the calculated cutoff still corresponded to a total of 167 CFU. This represents a clinically relevant bacterial load since it is known that less than 100 CFU can cause infection in the presence of implants [26].

The clinical relevance of these bacterial loads is also reflected in the diagnostic performance of MF. With respect to our results, low‐grade infections in presumed aseptic nonunion could be diagnosed by MF with a sensitivity of 64% and a specificity of 89%. Although the sensitivity of MF was, therefore, lower than that of SFC in this study, the specificity was increased by the CFU threshold, resulting in a 40% reduction in false‐positive results. Other studies investigating the diagnostic performance of sonication, restricted to cases of FRI, are limited. Those available studies have reported sensitivities between 52% and 91% and specificities between 71% and 93% [7, 9, 13]. When comparing MF using the calculated cutoff with the current standard methods of long‐term TC and histopathology performed in this study, the specificity of MF was comparable to both, while its sensitivity was inferior to that of TC but outperformed that of histopathology. Studies on the diagnostic performance of TC in FRI reported sensitivities between 60% and 89% and specificities between 71% and 100% [7, 8, 9, 10, 11]. The diagnostic performance of MF was, therefore, in the TC range. The HOES used to assess histopathological signs of infection in this study considers not only polymorphonuclear neutrophils (PMNs) infiltration but also osseous and soft‐tissue changes [22]. Compared to HOES, other FRI studies evaluating PMN quantification alone have demonstrated higher sensitivities ranging from 61% to 80% [10, 11, 12]. MF demonstrated a comparable level of performance in this present study with a sensitivity of 64%. Overall, the less‐than‐perfect performance of each diagnostic method in this study confirmed the approach of the FRI consensus definition that an infection can only be confirmed by the presence of at least two positive samples for the same pathogen [3, 23].

For treatment, however, it is important to know not only whether an infection is present, but also which microorganisms are causing the infection. Therefore, we compared the culture results of MF with those of the other microbiological methods. The microorganisms identified by MF were not statistically different from the bacterial spectrum of TC and SFC. CoNS and *C. acnes* were the most common bacterial species detected by all three methods. This is consistent with the results of a meta‐analysis of a systematic review by Wagner et al., who reported that 59% of surprise positive cultures in long‐bone nonunion were attributed to CoNS, followed by 15% to Cutibacterium [4]. MF, however, showed more polymicrobial detections than SFC and TC, although the difference was only statistically significant between MF and TC. The potential of implant sonication to increase the detection rate of polymicrobial infections in FRI has been speculated in previous studies [27]. In addition, differences in bacterial detection may also be due to the use of different culture media. While MF was conducted on solid media, SFC and TC were cultured in broth, in which isolation of bacterial strains is more difficult and potential competition for nutrients and limited space must be considered [28, 29]. From a clinical perspective, it is important to know all the pathogens involved to treat the infection with targeted antibiotics that are effective against bacterial species and their antibiotic susceptibilities. The current recommendation for systemic antimicrobial therapy in FRI is to initiate empiric antimicrobial treatment after revision surgery and to reassess antibiotics once microbiologic results are available [30, 31, 32]. Because of the high likelihood of low‐grade infections, every nonunion should be considered infected until proven otherwise [33]. However, infection can only be excluded, most likely by sterility of long‐term TCs after 14 days. MF offers a critical advantage over TC and SFC in that aerobic culture results are available in 2 days and anaerobic results in 5 days. Using an Anoxomat system to generate an anaerobic atmosphere, in which anaerobic bacteria are known to grow faster than in the gas‐pak method [34, 35], this shorter incubation time was sufficient to obtain countable, distinct colonies while minimizing the risk of neglecting otherwise slower‐growing anaerobic bacterial species. This approach is supported by a study by Jeverica et al. demonstrating a growth rate of 99% for *C. acnes* isolates from orthopedic implant‐associated infections on sheep blood agar under anaerobic conditions generated by an Anoxomat system, with a mean and standard deviation of time to detection of 54 ± 10 h [36]. Therefore, the MF method, in combination with TC, has the potential to be clinically relevant, as targeted antibiotic treatment should be initiated as early as possible [32]. Based on the results of this study, an early positive MF test result would indicate a 70% probability of infection. A negative result, on the other hand, would indicate an 86% probability of aseptic nonunion. This would allow targeted antibiotic therapy for all the pathogens involved to be initiated much earlier or discontinued if MF is negative and then adjusted as needed once long‐term TC results are available. This would contribute to a reduction in the use of broad‐spectrum or reserve antibiotics to slow down the ongoing development of resistance or to prevent its future development through targeted use.

The implementation of the MF method in microbiology laboratories for routine clinical diagnostics appears feasible and realistic. The laboratory protocol of the MF method, detailed in a previous publication [13], is characterized by its simplicity and ease of execution. In addition, MF is less susceptible to contamination and error. However, the processing time of MF, as well as the working space required in a laminar flow, should be taken into consideration. Importantly further validation of MF is necessary before this method may be implemented in routine clinical practice. In any future validation study, care should be taken to obtain five separate deep tissue samples for long‐term TC during nonunion revision procedures to allow for better comparison of MF and TC results. The study had some limitations. First, although the preoperative exclusion of suspected infection was based on the patient's history, clinical presentation, and blood counts, it was ultimately a subjective assessment by the treating surgeon. However, this reflects clinical practice. Second, the sample size for low‐grade infection was relatively small, with 22 individuals. However, the patient population was very homogeneous, and the lower and upper limits of the 95% CI of the AUC were reasonably close, indicating that the AUC value is likely accurate [37]. Third, a cutoff value of 11.1 CFU per 10 mL sonication fluid represents the mathematical cutoff, which a clinical laboratory could round to the nearest decimal point based on the volume of sonication fluid filtered. Finally, the results of this study cannot answer whether the additional bacterial species detected by MF were involved in the bacterial colonization of the implant at the nonunion zone and were, therefore, clinically relevant in the sense of a polymicrobial infection or whether these species simply colonized other parts of the implant. This needs to be investigated in further studies on the localization of bacterial species in implant colonization and in investigations on the reasons for discrepancies between MF, TC, and SFC in relation to the final diagnosis. However, targeted antibiotic treatment should consider all potentially involved bacterial species for treatment.

These limitations are countered by significant strengths of the study, namely the standardized sonication protocol, including a defined volume of initial fluid. Bacterial colonization of the osteosynthesis material was evaluated in a very homogeneous study population that included only femoral and tibial shaft nonunion. In addition, the entire implants were sonicated, making the CFU counts more comparable between patients and the calculated CFU cutoff more reliable. For diagnosis of infection, the full spectrum of intraoperative diagnostic methods was used in all patients: long‐term culture, histopathology, and sonication. Finally, the study design included a follow‐up period to minimize the likelihood of missing a low‐grade infection.

## 5 Conclusions

The results of this study suggest that MF may be a promising adjunct to long‐term TC for the identification of infection in suspected aseptic nonunion. After further successful validation of MF, its implementation in clinical diagnostics would have the advantage that patients with a low‐grade infected nonunion could receive the appropriate antibiotics within a few days after revision surgery and that patients with an aseptic nonunion could be weaned off postoperative empirical antibiotic therapy earlier. This would allow for faster initiation of tailored treatment in patients with nonunion and, in the face of antibiotic stewardship, would also contribute to targeted and sparing use of antibiotics.

## Author Contributions

K.T. contributed to the conceptualization and implementation of the multicenter study, the laboratory methodology, laboratory analyses, and statistical analyses and drafted the article. S.E. contributed to the conceptualization and implementation of the multicenter study and laboratory analyses. F.W., S.H., and the SAND Research Group contributed to patient recruitment and sample and data collection. S.H. and P.A. contributed to the study design, funding acquisition, and implementation and supervised the study. All authors revised critically for important intellectual content and approved the final manuscript.

## SAND Research Group

Septic Aseptic Nonunion Differentiation (SAND) Research Group—BG Unfallklinik Murnau: Matthias Militz, Simon Hackl, Ferdinand Weisemann, Katharina Trenkwalder, Sandra Erichsen, Tobias Hentschel, and Peter Augat; BG Klinik Tübingen: Heiko Baumgartner and Marie Reumann; BG Klinik Ludwigshafen: Georg Reiter and Holger Freischmidt; BG Unfallklinik Frankfurt: Matthias Kemmerer; BG Klinikum Bergmannstrost Halle: Steffen Langwald and John Hanke; BG Klinikum Duisburg: Martin Glombitza and Eva Steinhausen; BG Klinikum Hamburg: Ulf‐Joachim Gerlach; BG Klinikum Unfallkrankenhaus Berlin: Nikolai Spranger; BG Kliniken – Klinikverbund: Dirk Stengel.

## Conflicts of Interest

The authors declare no conflicts of interest.
